# Patterns in Food Insecurity During Pregnancy, 2004 to 2020

**DOI:** 10.1001/jamanetworkopen.2023.24005

**Published:** 2023-07-18

**Authors:** Stefanie N. Hinkle, Cara D. Dolin, Shimrit Keddem, Eliza W. Kinsey

**Affiliations:** 1 Department of Biostatistics, Epidemiology and Informatics, Perelman School of Medicine, University of Pennsylvania, Philadelphia; 2 Department of Obstetrics and Gynecology, Perelman School of Medicine, University of Pennsylvania, Philadelphia; 3 Department of Obstetrics and Gynecology, Ob/Gyn & Women’s Health Institute, Cleveland Clinic Lerner College of Medicine, Cleveland, Ohio; 4 Department of Family Medicine and Community Health, Perelman School of Medicine, University of Pennsylvania, Philadelphia; 5 Center for Health Equity, Research and Promotion, Corporal Michael J. Crescenz VA Medical Center, Philadelphia, Pennsylvania

## Abstract

This survey study assesses patterns in food insecurity during pregnancy among individuals in 14 US states participating in the Pregnancy Risk Assessment Monitoring System from 2004 to 2020.

## Introduction

Approximately 10% of US households are food insecure, meaning they experience limited or uncertain access to enough food for an active, healthy life.^1^ Moreover, nearly 4% experience very low food security, meaning that household members have reduced or disrupted eating due to lack of money or other resources. Adults with food insecurity (FI) are most likely to be female and of reproductive age.^2^ Food insecurity in pregnancy is understudied and may be associated with adverse pregnancy outcomes.^3^ The US Department of Agriculture (USDA) FI surveillance report does not distinguish by pregnancy status. The last population-based FI estimates were limited to California from 2002 to 2006.^4^ More recent population-based estimates on the burden of FI in pregnancy are lacking. We estimated population-based patterns of FI in pregnancy across 14 states from 2004 to 2020.

## Methods

This survey study was approved by the University of Pennsylvania Institutional Review Board with a waiver of informed consent due to the use of deidentified data. We analyzed data from individuals 18 years or older who delivered infants between January 2004 and December 2020 within 14 states participating in the Pregnancy Risk Assessment Monitoring System (PRAMS)^5^ (eMethods in Supplement 1). Participants were asked via questionnaire, “During the 12 months before your new baby was born, did you ever eat less than you felt you should because there wasn’t enough money to buy food?”

We estimated age- and state-adjusted yearly FI prevalence using the direct method, with data weighted to the 2020 distributions. We adjusted for age to account for any shifts in the age of pregnant people across the study period. We also adjusted for state because not all states consistently had data on FI across the study period or met the Centers for Disease Control and Prevention response rate threshold to be included in the data set. We tested for modification by age, race and ethnicity, and parity by including an interaction term with year. Data were weighted to account for the complex sampling design and nonresponse and to reflect population estimates. Analysis was performed using SAS software, version 9.4 (SAS Institute). The significance threshold was 2-sided *P* = .05.

## Results

Among 129 540 participants, most were ages 25 to 29 years (30.4%) and of non-Hispanic White race and ethnicity (68.3%). Prevalence of FI varied by individual characteristics and delivery year (Table). The adjusted FI prevalence was 8.9% (95% CI, 7.6%-10.1%) in 2004 and 6.7% (95% CI, 6.0%-7.5%) in 2020; FI fluctuated across years, with lower prevalence in 2006 (7.9%; 95% CI, 6.8%-9.1%) and higher prevalence in 2013 (10.4%; 95% CI, 9.1%-11.6%) (Figure). There were no interactions by age, race and ethnicity, or parity.

**Table.** Characteristics of the Study Population Overall and by Food Insecurity Status, 2004-2020^a^

Characteristic	Overall (N = 129 540)		Food secure (n = 117 002)^b^		Food insecure (n = 12 538)^c^^,^^d^^,^^e^	
	Unweighted No.	Weighted % (SE)	Unweighted No.	Weighted % (SE)	Unweighted No.	Weighted% (SE)
Age range, y^f^						
18-19	6224	4.5 (0.1)	5088	4.1 (0.1)	1136	8.5 (0.4)
20-24	27 676	20.6 (0.2)	23 175	19.1 (0.2)	4501	36.3 (0.7)
25-29	38 269	30.4 (0.2)	34 707	30.5 (0.2)	3562	29.5 (0.7)
30-34	35 811	28.5 (0.2)	33 662	29.6 (0.2)	2149	16.9 (0.6)
35-39	17 567	13.1 (0.1)	16 608	13.7 (0.2)	959	7.2 (0.4)
≥40	3993	2.8 (0.1)	3762	2.9 (0.1)	231	1.7 (0.2)
Self-reported race and ethnicity^f^^,^^g^						
American Indian or Alaska Native	5118	1.1 (0)	4284	1.0 (0)	834	2.0 (0.1)
Asian or Pacific Islander	7425	3.8 (0.1)	7067	4.0 (0.1)	358	1.7 (0.1)
Hispanic	23 470	16.5 (0.1)	21 022	16.2 (0.1)	2448	20.1 (0.6)
Non-Hispanic Black	10 668	7.2 (0.1)	9270	6.8 (0.1)	1398	10.9 (0.5)
Non-Hispanic White	76 274	68.3 (0.2)	69 655	69.0 (0.2)	6619	61.1 (0.7)
Other, unknown, or multiple races	6585	3.1 (0.1)	5704	3.1 (0.1)	881	4.1 (0.3)
Parity^f^						
0	53 173	39.4 (0.2)	48 197	39.6 (0.2)	4976	38.3 (0.7)
1	41 090	33.4 (0.2)	37 649	33.8 (0.2)	3441	28.7 (0.7)
2	20 666	16.3 (0.2)	18 402	16.1 (0.2)	2264	18.3 (0.6)
≥3	14 611	10.9 (0.1)	12 754	10.5 (0.1)	1857	14.8 (0.5)
Insurance^f^^,^^h^						
Medicaid	42 431	37.5 (0.2)	35 522	34.5 (0.2)	6909	68.5 (0.8)
Private, uninsured, or other	57 677	62.5 (0.2)	55 020	65.5 (0.2)	2657	31.5 (0.8)
Marital status^f^^,^^i^						
Married	83 777	66.2 (0.2)	78 946	68.8 (0.2)	4831	39.0 (0.7)
Other	45 575	33.8 (0.2)	37 909	31.2 (0.2)	7666	61.0 (0.7)
Educational level, y^f^^,^^j^						
<12	15 981	12.3 (0.2)	13 492	11.6 (0.2)	2489	19.8 (0.6)
12	32 573	24.9 (0.2)	27 735	23.5 (0.2)	4838	39.6 (0.8)
>12	80 008	62.8 (0.2)	74 932	64.9 (0.2)	5076	40.6 (0.8)
Year of delivery^k^						
2004	7242	4.1 (0)	6514	4.1 (0)	728	4.5 (0.3)
2005	7083	4.1 (0)	6425	4.1 (0)	658	4.2 (0.3)
2006	7334	4.3 (0)	6643	4.3 (0)	691	4.2 (0.3)
2007	7276	4.4 (0)	6506	4.3 (0)	770	5.0 (0.3)
2008	7787	6.0 (0)	6830	5.8 (0)	957	7.5 (0.4)
2009	7484	5.5 (0)	6692	5.4 (0)	792	6.2 (0.4)
2010	6868	3.8 (0)	6179	3.8 (0)	689	3.8 (0.2)
2011	6745	3.8 (0)	6083	3.8 (0)	662	3.5 (0.2)
2012	5162	5.7 (0)	4645	5.6 (0.1)	517	5.9 (0.4)
2013	7458	5.7 (0)	6689	5.6 (0)	769	6.6 (0.4)
2014	4245	3.5 (0)	3818	3.5 (0)	427	3.2 (0.3)
2015	7164	5.7 (0)	6523	5.7 (0)	641	5.5 (0.4)
2016	8094	8.1 (0)	7302	8.1 (0.1)	792	7.5 (0.4)
2017	8913	8.6 (0)	8048	8.7 (0)	865	8.3 (0.4)
2018	9608	9.3 (0)	8743	9.3 (0.1)	865	9.4 (0.5)
2019	10 840	9.0 (0.1)	9883	9.1 (0.1)	957	8.5 (0.4)
2020	10 237	8.5 (0)	9479	8.7 (0.1)	758	6.2 (0.3)

^a^ Among pregnant individuals in the 14 states participating in the Pregnancy Risk Assessment Monitoring System.

^b^ Represents 91.2% (95% CI, 90.9%-91.4%) of participants.

^c^ Represents 8.8% (95% CI, 8.6%-9.1%) of participants.

^d^ Distributions differed by food insecurity status across all participant characteristics (*P* < .001 based on χ^2^ analysis).

^e^ Food insecurity was assessed via the following question: “During the 12 months before your new baby was born, did you ever eat less than you felt you should because there wasn’t enough money to buy food?” This question is likely reflective of more severe food insecurity due to the presence of disrupted eating.

^f^ Characteristics were obtained from the birth certificate.

^g^ Race and ethnicity categories were self-reported on the birth certificate. Categorization was based on standard US Census categories, with the exception of Pacific Islander individuals, who could not be reported separately due to the small sample (n = 83) and were therefore combined with Asian individuals into a single category. The *other* category represents those who selected *other race* on the birth certificate or those who were categorized as *other non-Hispanic* individuals by Vermont because Vermont only reports race and ethnicity in 2 categories: *non-Hispanic White* and *non-Hispanic other*.

^h^ Data were missing for 29 432 participants.

^i^ Data were missing for 188 participants.

^j^ Data were missing for 978 participants.

^k^ Yearly estimates shown are for delivery year but are approximately reflective of the previous year due to the questionnaire time frame of the 12 months before the birth of the infant.

**Figure.**
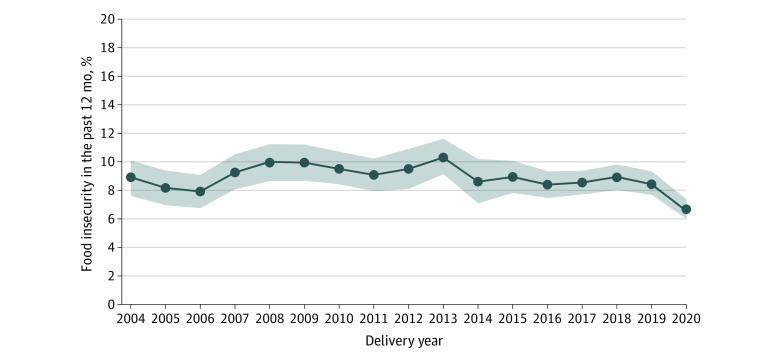
Patterns in Food Insecurity During Pregnancy, 2004-2020 Among pregnant individuals in the 14 states participating in the Pregnancy Risk Assessment Monitoring System. Yearly prevalence estimates shown for delivery year are approximately reflective of the previous year due to the questionnaire time frame of the 12 months before the birth of the infant. Estimates were adjusted for maternal age to account for any shifts in the age of pregnant people across the study period. Estimates were also adjusted for state because although the 14 participating states provided data on food insecurity, not all states consistently had data on food insecurity across the study period. All adjustments were completed using the direct method, with data weighted to the 2020 distributions. Shading represents 95% CIs.

## Discussion

Across 14 states, modest reductions in FI during pregnancy occurred across the study period, particularly since 2013. These findings parallel national FI patterns in the general population, whereby before the COVID-19 pandemic, a decrease occurred since 2011 (with return to prerecession levels).^1^ In 2020, 6.7% of individuals reported FI in the year before delivery. Based on questionnaire timing, these data are likely reflective of prepandemic FI. More research is needed to understand the implications of the COVID-19 pandemic for FI prevalence in pregnancy. The PRAMS FI prevalence is likely an underrepresentation of true FI prevalence in pregnancy given the question’s focus on disrupted eating, which typically reflects more severe FI.^6^ In 2020, national FI prevalence was 10.5%, and very low FI prevalence (including patterns of disrupted eating) was 3.9%.^1^ While direct comparisons between the PRAMS prenatal estimate and USDA national estimate are not possible, these rates suggest pregnant people may be disproportionately impacted by severe FI. This study is limited because the states included are not generalizable to the entire US, and FI is defined using a single question that likely reflects more severe FI.^6^ More research is needed on the association of FI with pregnancy outcomes and for strategies to alleviate FI among pregnant people.

## Notes

Supplement 1. **eMethods.** Study Sample Flowchart

Supplement 2.
Data Sharing Statement
