# Supplementary material for: Patterns in Food Insecurity During Pregnancy, 2004 to 2020
Source: JAMA Netw Open. 2023 Jul 18;6(7):e2324005. doi: 10.1001/jamanetworkopen.2023.24005 (PMC10354677; doi:10.1001/jamanetworkopen.2023.24005)
Supplement: Supplement 1. — eMethods. Study Sample Flowchart [file jamanetwopen-e2324005-s001.pdf]

Supplementary Online Content

Hinkle SN, Dolin CD, Keddem S, Kinsey EW. Patterns in food insecurity during pregnancy, 2004 to 2020. *JAMA Netw Open*. 2023;6(7):e2324005. doi:10.1001/jamanetworkopen.2023.24005

eMethods. Study Sample Flowchart

This supplemental material has been provided by the authors to give readers additional information about their work.

© 2023 Hinkle SN et al. *JAMA Network Open*.

eMethods. Study Sample Flowchart

Pregnancy Risk Assessment Monitoring System 2000-2020 (n=816,362)

- States that met the CDC threshold for reporting data

↓

Exclusions:

- States and Years where the food insecurity question was not asked

↓

Pregnancy Risk Assessment Monitoring System 2004-2020 (n=134,326)

- Colorado 2004-2013, 2015-2020
- Iowa 2013-2020
- Kansas 2017-2020
- Maine 2004-2020
- Minnesota 2009-2011
- Missouri 2016-2020
- New Mexico 2012-2020
- Oregon 2004-2013, 2015, 2018-2020
- Pennsylvania 2012-2020
- Tennessee 2008-2009
- Vermont 2004-2015
- Washington 2004-2008
- Wisconsin 2016-2020
- Wyoming 2016-2020

↓

Exclusions:

- Individuals < 18 years of age

↓

Pregnancy Risk Assessment Monitoring System 2004-2020 (n=131,686)

Exclusions:

- Individuals missing food insecurity (n=1702)
- Individuals missing age (n=5)
- Individuals missing race/ethnicity (n=0)
- Individuals missing parity (n=446)

↓

Pregnancy Risk Assessment Monitoring System 2004-2020 (n=129,540 – weighted to represent N=4,800,150)

© 2023 Hinkle SN et al. *JAMA Network Open*.
