# Supplementary material for: Patterns in Food Insecurity During Pregnancy, 2004 to 2020
Source: JAMA Netw Open. 2023 Jul 18;6(7):e2324005. doi: 10.1001/jamanetworkopen.2023.24005 (PMC10354677; doi:10.1001/jamanetworkopen.2023.24005)
Supplement: Supplement 2. — Data Sharing Statement [file jamanetwopen-e2324005-s002.pdf]

Data Sharing Statement

Hinkle. Patterns in Food Insecurity During Pregnancy, 2004 to 2020. *JAMA Netw Open*. Published July 18, 2023. doi:10.1001/jamanetworkopen.2023.24005

Data
Data available: No

Additional Information
Explanation for why data not available: We have obtained these data from PRAMS under a data sharing agreement and are not permitted to share the data outside of those on our agreement. Parties interested in the data should contact the CDC PRAMS office.
